# Pericardial effusion in oncological patients: current knowledge and principles of management

**DOI:** 10.1186/s40959-024-00207-3

**Published:** 2024-02-16

**Authors:** S. Mori, M. Bertamino, L. Guerisoli, S. Stratoti, C. Canale, P Spallarossa, I. Porto, P. Ameri

**Affiliations:** 1 Department of Internal Medicine, University of Genova, Genova, Italy; 2 IRCCS Ospedale Policlinico San Martino, Genova, Italy

**Keywords:** Pericardium, Cancer, Cardio-oncology, Tamponade, Pericardiocentesis, Management.

## Abstract

**Background:**

This article provides an up-to-date overview of pericardial effusion in oncological practice and a guidance on its management. Furthermore, it addresses the question of when malignancy should be suspected in case of newly diagnosed pericardial effusion.

**Main body:**

Cancer-related pericardial effusion is commonly the result of localization of lung and breast cancer, melanoma, or lymphoma to the pericardium via direct invasion, lymphatic dissemination, or hematogenous spread. Several cancer therapies may also cause pericardial effusion, most often during or shortly after administration. Pericardial effusion following radiation therapy may instead develop after years. Other diseases, such as infections, and, rarely, primary tumors of the pericardium complete the spectrum of the possible etiologies of pericardial effusion in oncological patients.

The diagnosis of cancer-related pericardial effusion is usually incidental, but cancer accounts for approximately one third of all cardiac tamponades. Drainage, which is mainly attained by pericardiocentesis, is needed when cancer or cancer treatment-related pericardial effusion leads to hemodynamic impairment. Placement of a pericardial catheter for 2-5 days is advised after pericardial fluid removal. In contrast, even a large pericardial effusion should be conservatively managed when the patient is stable, although the best frequency and timing of monitoring by echocardiography in this context are yet to be established. Pericardial effusion secondary to immune checkpoint inhibitors typically responds to corticosteroid therapy. Pericardiocentesis may also be considered to confirm the presence of neoplastic cells in the pericardial fluid, but the yield of cytological examination is low.

In case of newly found pericardial effusion in individuals without active cancer and/or recent cancer treatment, a history of malignancy, unremitting or recurrent course, large effusion or presentation with cardiac tamponade, incomplete response to empirical therapy with nonsteroidal anti-inflammatory, and hemorrhagic fluid at pericardiocentesis suggest a neoplastic etiology.

## Background

Pericardial effusion is quite a common finding in oncological patients. The diagnosis may be clinical, but most frequently relies on imaging techniques, especially transthoracic echocardiography (TTE) and computed tomography (CT), and treatment differs depending on the etiology and presentation.

In this narrative review article, we discuss the causes, manifestations, and management of pericardial effusion in cancer patients, with the goal of providing an up-to-date guidance for health care professionals. Furthermore, we address the question of when malignancy should be suspected in case of newly diagnosed pericardial effusion and no pre-exiting diagnosis of cancer.

### Risk factors for and causes of pericardial effusion in patients with cancer

#### Cancer

**Fig. 1:**
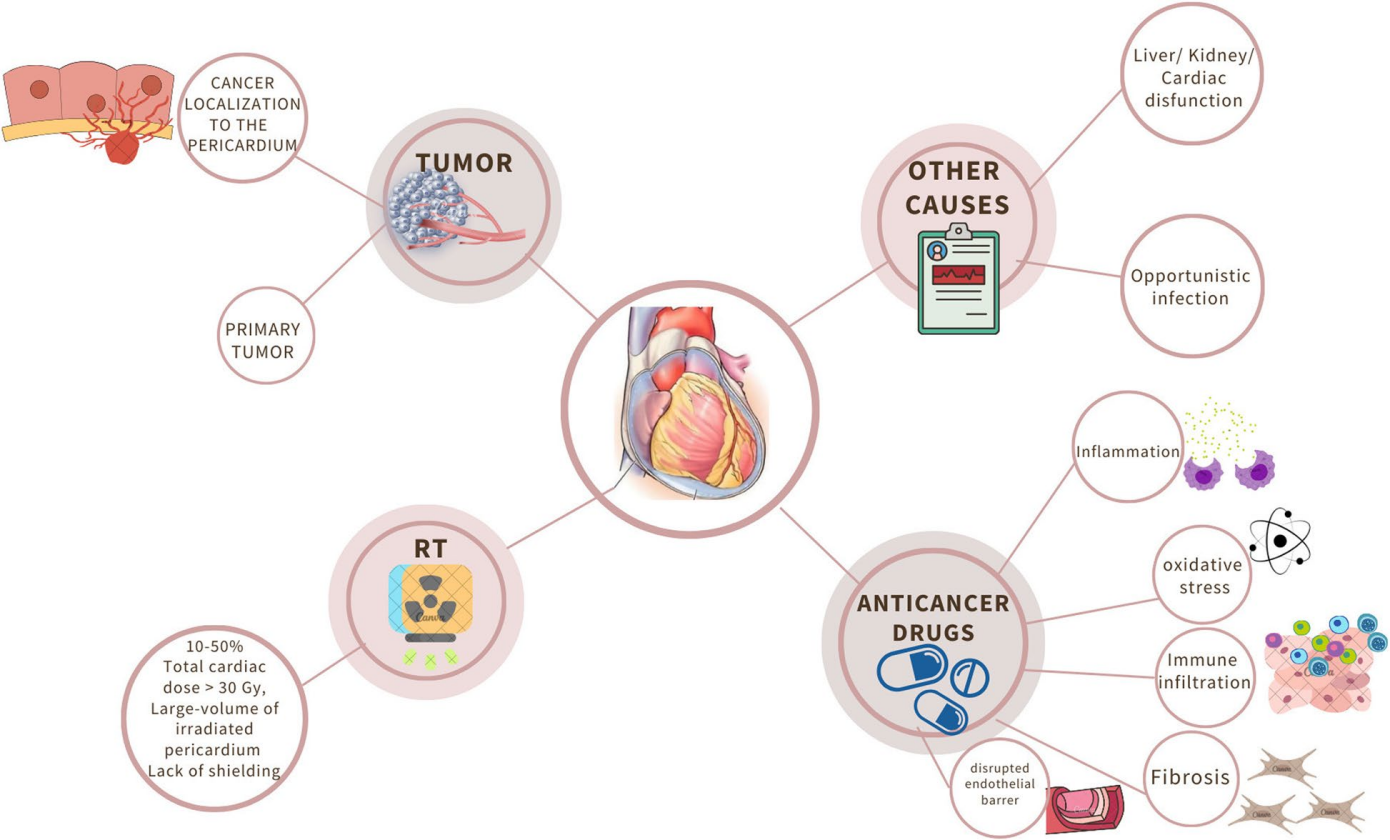
Causes of pericardial effusion in patients with cancer. Gy, gray; RT, radiation therapy

Cancer-related pericardial effusion is usually the consequence of secondary tumor localization to the pericardium [1] (Fig. 1). Although pericardial involvement may be the presentation of cancer, it is often found at advanced stages of disease and portends a poor prognosis [2, 3]. Neoplastic cells reach the pericardium by direct invasion, lymphatic dissemination, or hematogenous spread, disrupt blood flow or infiltrate the wall of pericardial capillary and small veins, and elicit the accumulation of transudate or blood into the pericardial space [4]. Lung cancer is the most frequent cause of metastatic pericardial effusion, also defined as malignant pericardial effusion, followed by breast cancer, esophageal cancer, pancreatic cancer, melanoma, and hematological malignancies, especially B-cell lymphoma [2, 3, 5, 6]. Less commonly, tumoral invasion of mediastinal lymph nodes results in pericardial effusion, because of obstruction of lymphatic drainage of the pericardium with formation of pericardial transudate [4].

Primary tumors of the pericardium are 100-1,000 times less frequent than secondary ones, with the prevalence ranging from 0.001 to 0.007% [7, 8]. Within this group of rare tumors, mesothelioma and lymphoma predominate. The vast majority of primary cardiac lymphomas are B-cell derived, particularly diffuse B-cell lymphoma; in immunocompromised individuals, virus-associated lymphomas can be found, such as primary effusion lymphoma, Burkitt lymphoma, and Epstein-Barr virus-related lymphoproliferative disorders [9].

#### Radiation therapy

Radiation therapy (RT) may cause acute pericardial effusion or lead to delayed pericardial effusion, developing months to decades following treatment, with an estimated rate of 10%, but as high as 50% in certain patient subsets [10, 11]. Late RT-related pericardial effusion is typically observed in subjects with younger age at cancer diagnosis and longer disease-specific survival. Pericardial injury occurs when RT is delivered to the chest and the heart is within the radiation field, as it can happen for mediastinal lymphoma or cancer of the breast, lung, and esophagus [11, 12]. Since breast cancer and Hodgkin lymphoma are relatively common and have better prognosis than other thoracic malignancies treated with RT, survivors from these types of tumors are at greatest risk of RT-initiated pericardial effusion [13].

Pericardial effusion secondary to RT is fibrin-rich or hemorrhagic and is thought to be the result of microvascular damage from ionizing radiation, which alters venous and lymphatic drainage from the pericardium, and radiation-elicited pericardial inflammation. The risk of pericarditis rises from 5% to more than 50% with the total dose of radiation increasing from 40 to 50 Gy (Gy) [14], and if more than 30% of the cardiac area receives 50 Gy [15]. However, it must be noted that current RT schemas aim at minimizing the collateral irradiation of the heart.

Risk factors for RT-induced pericardial effusion can be categorized in RT-related and patient-related. The former are total radiation dose to the heart > 30 Gy, large-volume of irradiated pericardium, and lack of radiation shielding [11–13]. Patient-related factors are older age, pre-existing cardiovascular risk factors, such as diabetes and obesity, and left-sided tumor. In addition, RT enhances the cardiotoxicity of certain anticancer drugs, including anthracyclines, platinum-based agents, taxanes, gemcitabine, bevacizumab, and immune checkpoint inhibitors (ICIs). In a retrospective analysis of data from 14,358 adult survivors of various childhood and adolescent cancers, pericardial effusion was independently associated with exposure to an anthracycline dose of 250 mg/m^2^ or more and with exposure to any dose of cyclophosphamide [16].

The guidelines on cardio-oncology of the European Society of Cardiology (ESC) state that TTE should be performed every 5 years in cancer survivors who received a mean heart dose (MHD) of ionizing radiation > 15–25 Gy, or between 5 and 15 Gy together with doxorubicin ≥ 100 mg/m2 or equivalent. If the MHD was > 15–25 Gy, or > 5–15 Gy in combination with doxorubicin ≥ 100 mg/m2, it is recommended to perform TTE 1, 3, and 5 after completion of cardiotoxic cancer therapy and every 5 years thereafter (class of recommendation IIa) [17].

#### Oncological medical therapy

Anti-cancer drugs potentially causing pericardial effusion include anthracyclines, cyclophosphamide, cytarabine, busulfan, tyrosine kinase inhibitors (TKI) used for treatment of chronic myelogenous leukemia (ponatinib, dasatinib, bosutinib and, to a smaller extent, nilotinib), arsenic trioxide, all-trans retinoic acid, and interleukin-2 [18–21].

The underlying mechanisms are multiple (Fig. 1). Anthracyclines, cyclophosphamide, cytarabine, and bleomycin trigger acute pericarditis, while many of the aforementioned medications may initiate the production of oxygen free radicals with ensuing oxidative stress. Other events accounting for the pathogenesis of cancer treatment-related pericardial effusion (CTR-pericardial effusion) are pericardial and endomyocardial fibrosis (busulfan) [21] and increased endothelial permeability (interleukin-2 and dasatinib) [22, 23].

Pericardial effusion has also been increasingly reported after initiation of ICIs. In a retrospective comparison of > 2,500 consecutive patients who received ICIs with age- and cancer-type matched controls who did not receive ICIs, the risk of pericarditis or pericardial effusion was more than fourfold higher in the ICI group after adjusting for potential confounders [24]. Pericardial effusion following ICIs may be due to increase in size of pericardial micro-metastases because of T cell infiltration (so-called pseudoprogression) [25]. Indeed, pericardial effusion generally occurs soon after ICI administration, with the median time to onset being 30 days [26]. It is remarkable that pericardial effusion upon ICI administration has mainly been described in subjects with lung cancer [26]. A possible explanation is that these patients are often also treated with RT and that ionizing radiation exposes pericardial antigens, which are then recognized by T lymphocytes [27].

The anti-PD1 antibody, nivolumab, has particularly been associated with significant pericardial effusion [26–28], although this observation may be flawed by a reporting bias. In case studies, cytological examination of pleural fluid and biopsy specimens of pericardial leaflets revealed T-lymphocyte infiltrates, mostly CD4+, in the absence of malignant cells, supporting the hypothesis of an autoimmune process driving the development of pericardial effusion under ICIs [28, 29]. Nevertheless, malignant cells were detected in pericardial fluid from about half of patients with pericardial effusion during ICI therapy [26].

#### Other factors and comorbidities

Pericardial effusion is more frequent in males than females with the ratio being 1.3:1. It may be the consequence of heart failure, liver dysfunction with hypoalbuminemia, and renal failure, either concomitant or caused by cancer or cancer treatment [18, 30, 31]. Several other comorbidities and conditions can lead to pericardial effusion in cancer patients, such as pneumonia/empyema and connective tissue disease, as well as thoracic procedures and interventions [32]. Opportunistic infections favored by immunosuppression, such as by cytomegalovirus, tuberculosis mycobacteria, and fungi, may elicit pericarditis with pericardial effusion [33]. Finally, antithrombotic therapies can determine pericardial bleeding [34, 35].

In retrospective cohorts with severe pericardial effusion requiring drainage, the frequency of pericardial effusion not attributed to cancer or cancer treatment was 42–58% [36, 37].

### Approach to the patient with cancer and pericardial effusion

**Fig. 2:**
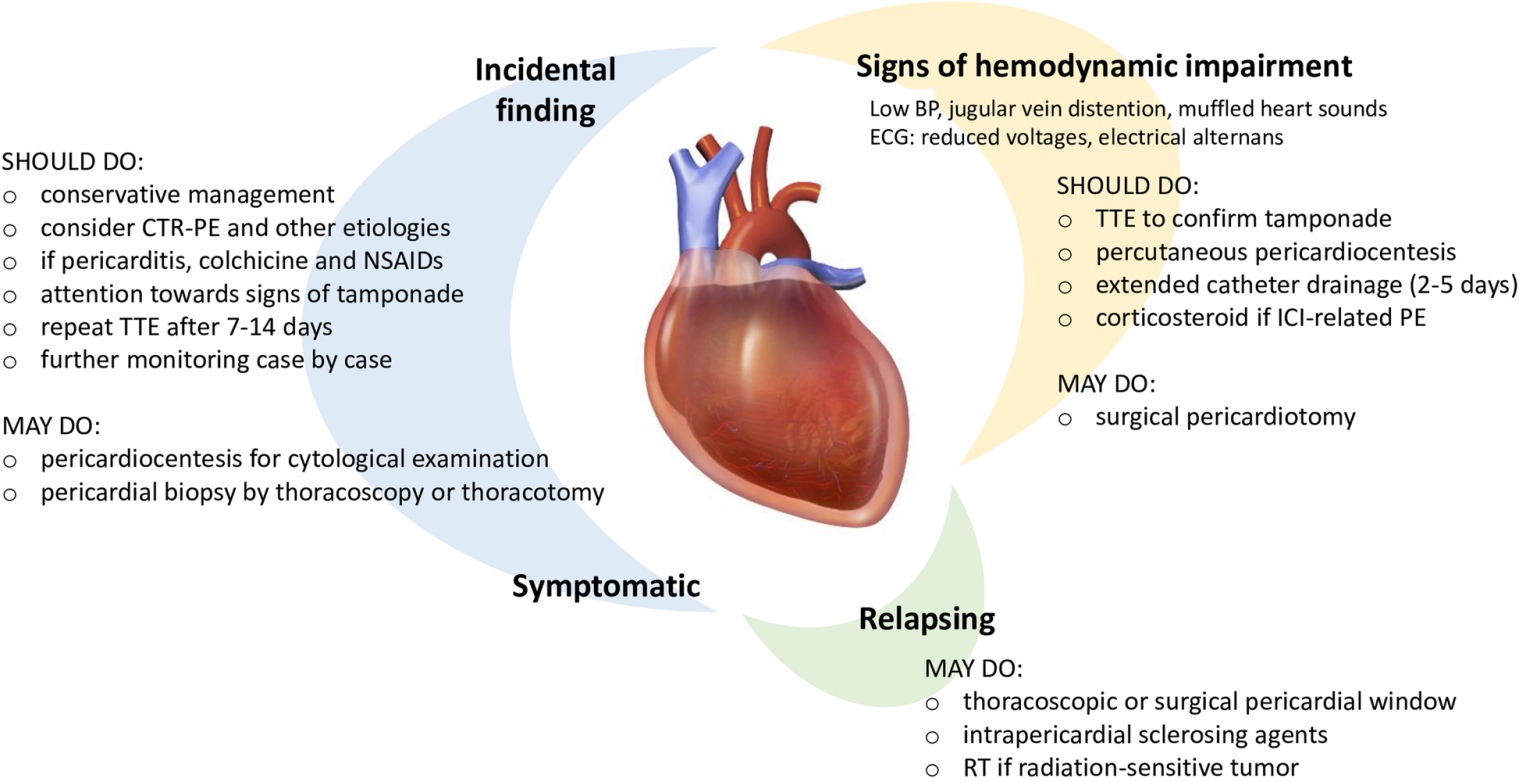
Management of large pericardial effusion in patients with cancer.  BP, blood pressure; CTR, cancer treatment-related; ICI, immune checkpoint inhibitor; NSAIDs, non-steroidal anti-inflammatory drugs; PE, pericardial effusion; RT, radiation therapy; TTE, transthoracic echocardiography.  The picture in the middle of the figure was obtained from Wikimedia Commons

The management of pericardial effusion in oncological patients varies depending on the cause and the clinical presentation [38]. The most challenging scenarios are when pericardial effusion is the reason for which the patient comes to medical attention and when pericardial effusion impairs cardiac hemodynamics (Fig. 2).

Anytime there is a history of cancer treatment known to induce pericardial effusion, the possibility of CTR toxicity should be considered. Timing of exposure is important, since onset of CTR-pericardial effusion can be during or years after antitumor therapy, as it occurs with ICIs and RT, respectively. Some drugs also cause pleural effusion, e.g. dasatinib [39].

Fever and weight loss can be manifestations of primary cardiac lymphoma, albeit this tumor is very rare.

The mediastinal connective tissue opposes relatively low resistance to the expansion of the external (parietal) pericardium, allowing the pericardial space to contain a large amount of fluid as long as it progressively accumulates. This explains why large pericardial effusion can develop in the absence of symptoms and signs. In contrast, adaptation is not possible when fluid rapidly accumulates in the pericardial space. Consequently, the pressure in the pericardial space suddenly increases up to compressing the right chambers of the heart, which have lower pressures than the left ones. This pathophysiology (pericardial tamponade) impairs right and, thereby, left cardiac filling, leading to low cardiac output and shock. The amount necessary for compression of the cardiac chambers may be as little as 250 mL if fluid quickly occupies the pericardial space [40, 41].

Abrupt or worsening pericardial effusion is generally symptomatic for shortness of breath, cough, or chest pain and should prompt the suspicion of metastatic localization to the pericardium. If cancer has not been diagnosed yet, the malignancies most often invading the pericardium should be carefully searched: lung and breast cancer, melanoma, and lymphoma [42].

When newly found pericardial effusion is moderate to severe, it is critical to pay attention to a drop in blood pressure and to the appearance of jugular vein distention (due to congestion transmitted backwards from the compressed right atrium), as these may be signs of impending pericardial tamponade.

When pericardial effusion is very large, ECG voltages are low in approximately 50% of patients, and electrical alternans can result from swinging of the heart within the pericardium [43].

TTE is key to confirm pericardial effusion, as well as to detect cardiac tamponade by showing enhanced ventricular interdependence, right atrial systolic collapse, right ventricular early diastolic collapse, and plethora of the inferior vena cava [44]. Among other imaging techniques, chest radiography may provide nonspecific cues: enlarged cardiac silhouette in the case of tamponade and “water bottle” appearance in the presence of chronic, abundant pericardial effusion; and pleural effusion and pulmonary infiltrates as additional findings [45]. CT also demonstrates pericardial effusion and is the best imaging modality to assess pericardial thickness and calcification [46], while cardiac magnetic resonance imaging (CMR) can reveal adhesions of the thickened pericardium to the myocardium of the left and right ventricles [47, 48].

Pericardial effusion is often incidentally discovered in subjects with an established cancer diagnosis, who undergo TTE or CT imaging for other reasons. The TTE criteria to classify the severity of pericardial effusion are summarized in Table 1. TTE re-evaluation of pericardial effusion that is at least moderate at first detection is reasonable (after 7–14 days according to the ESC guidelines on cardio-oncology [17]), but the cost-effectiveness of prolonged interval monitoring remains unclear (Fig. 2).

**Table 1:** Classification of pericardial effusion

Classification	Size	Volume of effusion
Trivial	Seen only during systole	< 50 mL
Small	< 1 cm	50–100 mL
Moderate	1–2 cm	100–500 mL
Large	> 2 cm	> 500 mL
Very large	> 2.5 cm	

The table is based on the recommendations of the American Society of Echocardiography for multimodality imaging of patients with pericardial disease, endorsed by the Society for Cardiovascular Magnetic Resonance and Society of Cardiovascular Computed Tomography (J Am Soc Echocardiogr. 2013;26:965–1012)

The size of pericardial effusion is measured as the maximum diameter perpendicular to the ventricular wall in diastole. The reported volume of effusion is approximate

Primary tumors of the pericardium can present with constrictive physiology. The neoplasm encasing the heart can be revealed by TTE, along with the pericardial effusion [49]. The imaging features on CT and CMR overlap with inflammatory pericarditis and include diffuse pericardial thickening and nodular enhancement.

About 30% of cardiac tamponades are due to malignancy [50], but the frequency by which, once diagnosed, cancer-related pericardial effusion progresses to tamponade appears to be low in clinical practice. In a recent investigation, 18,847 (0.1%) of 19,773,597 ≥ 18 year old patients in the US National Inpatient Sample discharged with a cancer code between January 2004 and December 2017 underwent pericardiocentesis [51]. Moreover, the prevalence of CTR-pericardial effusion requiring pericardiocentesis was reported to be 0.38%, 0.24%, 0.22%, and 0.20% in patients receiving ICIs, antimetabolites, TKIs, and monoclonal antibodies, respectively, and less than 0.14% with other therapies [26].

Cardiac tamponade is promptly resolved by percutaneous pericardiocentesis via subxiphoid or apical approach [28, 52]. The procedure can be done under the guide of TTE, fluoroscopy, or CT. TTE and fluoroscopy the choice in emergency situations. CT is a valuable alternative when the patient is stable [53]. Hemodynamically relevant pericardial effusion can also be removed by surgical pericardiotomy, but pericardiocentesis has less complications [17]. On the other hand, the need for repeat procedures may be greater if pericardial effusion is drained by pericardiocentesis [54]. The reported risk of infection with pericardiocentesis is 0.3% [55].

Pericardial drainage is usually not necessary in the absence of hemodynamic impairment [56]. Nevertheless, pericardiocentesis with cytological examination may be carried out to establish the etiology of the pericardial effusion, and to steer the diagnostic workup towards other causes if cancer cells are not found. It must be stressed that pericardiocentesis yields positive cytology in less than 50% of cancer-related pericardial effusions [57–59]. A frankly hemorrhagic pericardial fluid suggests that pericardial effusion is secondary to cancer metastases or primary angiosarcoma [9, 49]. Histopathologic evaluation of pericardial biopsy specimens collected by means of thoracoscopy or thoracotomy is another option to determine the etiology of pericardial effusion, but it is seldom followed [56].

A pericardial catheter may be left after pericardiocentesis for some days to promote adherence of pericardial layers, until minimal (< 30 mL/24 hours) or no liquid is drained [55, 59]. This strategy has been performed in patients with a platelet count < 50,000/µl without apparent increased risk of procedure-related bleeding and may reduce the risk of recurrence [36]. Experts advocate that a pericardial catheter is left for 2–5 days after drainage of cancer-related pericardial effusion [60]. However, no data are available about the risk of infection with prolonged pericardial catheter.

Creation of thoracoscopic or surgical pericardial window, usually into the pleural space, is another mean to prevent relapse of pericardial effusion [61, 62]. Palliation of pericardial effusion may also be achieved by intrapericardial instillation of cytotoxic/sclerosing agents or RT of radiation-sensitive tumors [52].

Pericardiectomy is the best treatment modality for primary pericardial tumors [55].

Drainage of pericardial effusion secondary to ICI therapy is rarely needed, since it normally responds to transient interruption of ICIs and corticosteroids [17, 26].

Pericardial effusion in the setting of post-actinic acute pericarditis is generally benign and does not impose to stop cancer therapy [49].

In case of clinically significant CTR-pericardial effusion, multidisciplinary discussion between the cardiologist and the oncologist/hematologist is fundamental to decide whether cancer treatment can be safely continued. Rechallenge with ICIs after pericardial effusion resolution appears to feasible [63].

Finally, pericarditis with pericardial effusion occurring in oncological patients, but unrelated to cancer or its treatment, should be managed like in the general population with colchicine and non-steroidal anti-inflammatory drugs [49]. Pericardiocentesis is indicated for inflammatory pericardial effusion that is unresponsive to treatment or symptomatic and moderate to large, and in the suspicion of bacterial or fungal infection. Pericardiectomy or a pericardial window may be considered if clinically relevant pericardial effusion recurs [49].

### Approach to the general patient with pericardial effusion: can it be cancer-related?

Although pericardial involvement is usually found in patients who have already been diagnosed with cancer, pericardial effusion can be the initial presentation of an occult malignancy, especially when it is large [32, 57, 58].

A study conducted in Denmark demonstrated that 1,550 (11%) patients out of 13,759 with pericarditis/pericardial effusion received a new cancer diagnosis during a median follow-up period of 6.4 years. Moreover, mortality was increased after cancer diagnosis [32].

Other studies also examined the risk of subsequent cancer among patients with a first-time diagnosis of pericarditis and showed that malignancy, most often pulmonary, of the breast or hematological, eventually underlied pericarditis/pericardial effusion in 12-23% of individuals [32, 64, 65].

Surprisingly, many of the clinical features believed to help to distinguish between different etiologies of pericardial effusion overlapped between cancer-related and other-etiology pericardial effusion. Fever and precordial pain, usually considered clinical markers of inflammation, were as common among patients with cancer-related pericardial effusion as among those with other causes of pericardial effusion [57].

Clinical factors that may indicate a neoplastic origin of pericardial effusion are a history of malignancy, unremitting or recurrent course, presentation with pericardial tamponade, and incomplete response to empirical therapy with nonsteroidal anti-inflammatory drugs [2, 32, 57, 58].

In the aforementioned Danish study, a history of heart failure, chronic obstructive pulmonary disease, alcohol-related diagnoses, tuberculosis, and recent pneumonia or empyema were also associated with elevated cancer standardized incidence ratios among patients with pericarditis. In contrast, the risk of cancer diagnosis was lower in subjects with than without recent thoracic surgery or myocardial infarction [32].

Normal levels of inflammatory biomarkers and hemorrhagic fluid at pericardiocentesis support the suspicion of malignant pericardial effusion [57, 64].

Cellularity and protein content of pericardial fluid were similar in pericardial effusion secondary to cancer versus heart failure or uremia. However, malignant pericardial effusion had lower glucose concentrations and higher lactate dehydrogenase concentrations [64].

We suggest starting a diagnostic work-up for malignancies in patients with cardiac tamponade, as well as moderate-to-severe pericardial effusion of unexplained origin not responding to conventional anti-inflammatory therapy.

## Conclusions

Cancer and cancer treatment deserve attention as possible causes of pericardial effusion, even in the general population of patients presenting with a new pericardial effusion. Although urgent and aggressive treatment is mandatory when there is hemodynamic instability, management of cancer-related and CTR-pericardial effusion should be conservative in most cases. Given the variety of causes and therapeutic and prognostic implications, interaction between health care professionals with cardiological and oncological/hematological expertise and case-by-case discussion are key to optimize the approach to the patient with pericardial effusion and cancer.

## Data Availability

No datasets were generated or analysed during the current study.

## Abbreviations

CMR: Cardiac magnetic resonance
CRT: Cancer treatment-related
CT: Computed tomography
ESC: European Society of Cardiology
Gy: Gray
ICIs: I mmune checkpoint inhibitors
MHD: Mean heart dose
RT: Radiation therapy
TKI: Tyrosine kinase inhibitors
TTE: Transthoracic echocardiography
